# Effect of water stress on growth of three linseed (*Linum usitatissimum* L.) varieties

**DOI:** 10.1186/s40064-016-2348-5

**Published:** 2016-06-17

**Authors:** Lilian Wambui Kariuki, Peter Masinde, Stephen Githiri, Arnold N. Onyango

**Affiliations:** Department of Horticulture, Jomo Kenyatta University of Agriculture and Technology, Juja, Kenya; School of Agriculture, Meru University of Science and Technology, Meru, Kenya; Department of Food Science and Technology, Jomo Kenyatta University of Agriculture and Technology, Juja, Kenya

**Keywords:** Available soil water, Drought tolerance, Number of leaves, Number of tillers, Plant dry weight, Plant height, Relative water content

## Abstract

Linseed (*Linum usitatissimum* L.) is an annual oil crop that accounts for approximately 1 % of the world’s oilseed supplies. It produces seeds that are rich in the health-promoting ω-3 fatty acid, α-linolenic. In Kenya, linseed is grown in the Rift Valley and Western regions, places which often experience drought. This study was aimed at evaluating the effect of water stress on growth of three linseed cultivars and to establish the extent of drought tolerance in the three cultivars. A greenhouse pot experiment in a completely randomized design was conducted at Jomo Kenyatta University of Agriculture and Technology, Kenya. The pots were well watered until the fourth week when watering was completely withheld to a half of the pots (stressed) while the other half (well watered control) was maintained at 90 % field capacity. Destructive harvesting was done when the stressed pots were at 90, 70, 60, 50, 40 % field capacities and at permanent wilting point. The experiment was replicated thrice and was repeated twice (February–May and August–November 2014). There were no significant differences in production of leaves, plant height, number of tillers and biomass between the three varieties in both seasons. Subjecting the linseed varieties to permanent wilting resulted in reduced production of leaves, growth in height, production of tillers and dry weight by 20–40 %. Decline in all growth parameters begun when 30–80 % of available soil water had been used up. There existed linear relationships between the various evaluated growth parameters. These relationships were not influenced either by the water status of soil or the varieties. Relative water content for the three linseed varieties declined after 25–67 % of available soil water had been used up.

## Background

Linseed (*Linum usitatissimum* L.) has been a major source of industrial oil for products like paints, linoleum, polish, inks and cosmetic (Green and Marshall 1984; Zhang et al. 2007). Currently, linseed is important as a functional food from the point of view of its nutrition and pharmaceutical value, and its nutritious components include oil, protein, lignin, resolvable fiber, mineral and vitamins (Wu et al. 2008). Notably, linseed is the best source of the n-3 fatty acid, α-linolenic acid (ALA), which constitutes nearly 55 % of its total fatty acids. This percentage is 5.5 times more than the next best sources of α-linolenic acid (Bloedon and Szapary 2004). ALA is an essential fatty acid which can be metabolized to eicosapentaenoic acid (EPA) and docosahexaenoic acid (DHA) by elongases and desaturases in humans (Chen et al. 2002). It is well known that ALA increases the absorption of long chain-polyunsaturated fatty acids (LCPUFA), especially EPA and DHA, and decreases the risks of physiological disorders such as colon tumor (Dwivedi et al. 2005), breast cancer (Chen et al. 2006; Thompson et al. 1996) and atherosclerosis (Prasad 1997; Yamashita et al. 2005).

In Kenya, linseed is grown in the Rift valley and Western Kenya regions (Riungu 1988), regions which often experience drought conditions. Several varieties of linseed exist at the National Plant Breeding Station in Njoro (Riungu 1988). These are however largely not characterized in terms of their response to varying growth conditions (Personal communication). There is therefore need to evaluate the factors that affect linseed production. Diepenbrock et al. (1995) reported high genotype-environment interactions in Europe, with yields varying considerably between seasons and locations. Nematallahi and Saeidi (2011) found significant differences in the response of several linseed genotypes to drought, with some being drought tolerant and others being drought sensitive.

Plants are often exposed to various environmental stresses under both natural and agricultural conditions. Drought stress is one of the most important environmental stresses limiting growth and productivity of plants. Drought can significantly influence plant performance and survival and can lead to major constraints in plant functioning, including a series of morphological, physiological and metabolic changes (Fisher and Maurer 1978; Ludlow and Muchow 1990). Drought affects photosynthesis directly and indirectly and consequently dry matter production, and its allocation to various plant organs (Mayaki et al. 1976). Drought stress also reduces leaf expansion and production, and promotes senescence and abscission (Karamanos 1980).

Ahmad et al. (2007) reported a 39 % dry matter reduction in wheat when water levels were reduced from 90 to 30 %FC. Ramos et al. (1999) reported inhibited accumulation in fresh plant mass by 88 % compared to dry biomass (85 %) when moisture levels dropped from 100 to 30 %FC for cv. EMGOPA-201. This relatively lower influence of drought on dry biomass than on fresh mass signified a presence of disturbances in water relations. Lazcano-Ferrat and Lovatt (1999) reported a decrease of 14–27 % in dry biomass in young bean plants subjected to drought and significant increase in ratio dry mass/fresh mass (DM/FM). It is considered that increased ratio DM/FM is a stress parameter at plant level (Baker 1993; Augé et al. 2001).

The capacity to maintain high relative water content (RWC) values under drought was reported in drought tolerant bean cultivars (Zlatev 2005) and in *Astragalus gombiformis* Pom. and *Medicago sativa* L. (Gorai et al. 2010). For bean plants this could be explained by their ability to accumulate great quantities of proline and other osmotic active compounds which are involved in the reduction of osmotic potential and in osmotic adjustment (Zlatev 2005).

Linseed is drought tolerant (Nematallahi and Saeidi 2011). However genotype–environment interactions have been shown to be high for linseed (Diepenbrock et al. 1995), and yields vary considerably between seasons, depending on location and weather. The objective of this study was to evaluate the effect of water stress on different growth parameters and the relative water content of three linseed varieties, namely Summit, S19/12 and Raja.

## Results

**Fig. 1:**
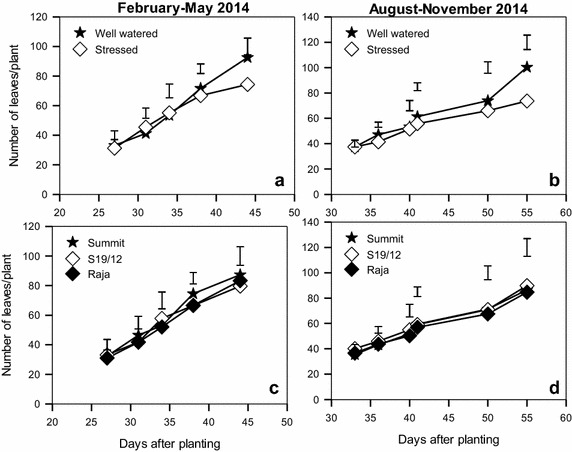
Production of leaves by linseed varieties (**c**, **d**) grown under water stress (**a**, **b**) during the periods February–May and August–November, 2014. *Vertical bars* show LSD_0.5_

The soil that was used for this experiment had a pH of 6.2, electrical conductivity of 0.12 dS/m which are within the normal range for most crops (Okalebo et al. 2002). Production of leaves by well watered and stressed plants became significantly different (P < 0.05) from 40 days after planting (Fig. 1). The well-watered produced 92 and 100 leaves during the February–May and August–November 2014 seasons respectively (Fig. 1a, b). The stressed plants produced 74 leaves during each of the two seasons. However, there were no significant differences in the three varieties’ production of leaves in response to moisture levels in both seasons (c, d).

**Fig. 2:**
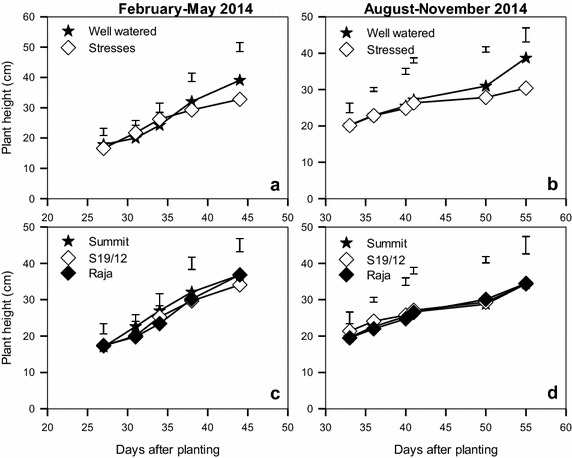
Effect of water stress (**a**, **b**) on plant height of linseed varieties (**c**, **d**) during the periods February–May and August–November, 2014. *Vertical bars* show LSD_0.5_

The well watered and stressed plants did not vary significantly in height until after 40 days after planting. Well watered plants grew to heights of 39 cm while stressed plants reached 31 cm (Fig. 2a, b). For the three tested linseed varieties, plant height was not significantly influenced by moisture level (Fig. 2c, d). Summit and Raja were taller than S19/12 during the February–May season while all the varieties attained similar heights of 34 cm in the August–November season.

**Fig. 3:**
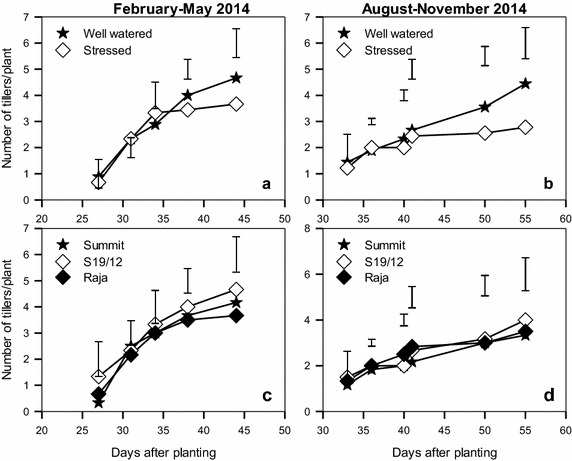
Mean number of tillers produced by linseed varieties (**c**, **d**) grown under water stress (**a**, **b**) during the periods February–May and August–November, 2014. *Vertical bars* show LSD_0.5_

In both February–May and August–November 2014 seasons, the number of tillers was higher in well watered plants from 40 days after planting (Fig. 3a, b). The difference was significant only during the August–November season (P < 0.05). The well watered plants produced 4–5 tillers compared to 3–4 tillers in stressed plants. The three linseed varieties did not differ significantly in production of tillers in both seasons (Fig. 3c, d). In both seasons, S19/12 produced the highest number of tillers with 4–5 tillers, while the least number of tillers was produced by Raja in both seasons with 2–3 tillers.

**Fig. 4:**
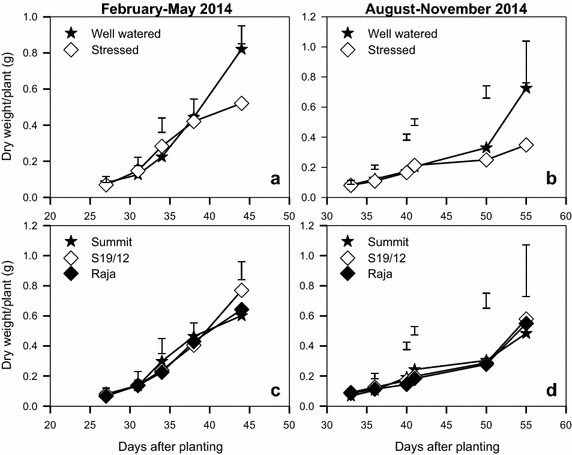
Dry weight accumulation by linseed varieties (**c**, **d**) grown under water stress (**a**, **b**) during the periods February–May and August–November, 2014. *Vertical bars* show LSD_0.5_

There was significant difference in dry weight between well watered and stressed plants beyond 40 days after planting in both seasons (Fig. 4). Well watered plants produced significantly higher dry weight ranging 0.73–0.82 g/plant compared to 0.35–0.52 g/plant for stressed plants (Fig. 4a, b). In the February–May 2014 season, S19/12 gave significantly higher dry weight (0.77 g) (Fig. 4c) compared to Raja (0.64 g) and Summit (0.60 g) (P < 0.005) from 45 days after planting. However, in the August–November season, the three cultivars did not differ significantly in dry weight even at day 55, although S19/12 still gave higher dry weight than the other two varieties (Fig. 4d).

**Fig. 5:**
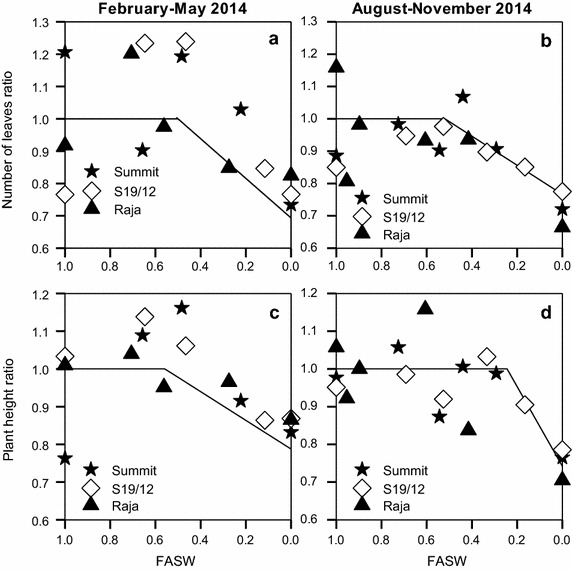
Ratio of number of leaves (**a**, **b**) and plant height (**c**, **d**) of stressed plants to well watered plants in response to changing fraction of available soil water (FASW) for linseed varieties grown during the periods February–May and August–November, 2014

Decline in production of leaves in both seasons started at fraction of available soil water (FASW) of 0.5 (Fig. 5a, b; Table 1). The decline was similar for the three varieties. Production of leaves however ceased at a point when the ratio of leaves of the stressed plants to the well watered was 0.6–0.8.

**Table 1:** Relationship between slope and fraction of available soil water critical points (FASW_c_) and their 95 % confidence intervals (CI) for the non-linear functions in Figs. 5, 8 and 10

	February–May 2014				August–November 2014			
	Slope	95 % CI	FASW_c_	95 % CI	Slope	95 % CI	FASW_c_	95 % CI
Leaves	0.29	0.2873–0.3075	0.50	0.4887–0.5209	0.72	0.7161–0.7363	0.52	0.4982–0.5391
Height	0.38	0.3783–0.3996	0.56	0.5579–0.5602	0.70	0.6250–0.9681	0.20	0.1707–0.3707
Dry weight	1.17	1.1647–1.1769	0.43	0.4267–0.4375	0.81	0.8111–0.8245	0.62	0.6104–0.6319
%RWC	1.66	1.6517–1.6753	0.33	0.3229–0.3433	1.26	1.2509–1.2738	0.75	0.7357–0.7646

During the February–May season, decline in plant height for the stressed plants begun at FASW of 0.56. This decline however started at 0.20 FASW during the August–November season (Table 1). Severe stress caused plants to cease increasing in height. In both seasons, this happened when the ratio of the stressed plants was 0.7–0.8 that of the well watered plants (Fig. 5c, d).

**Fig. 6:**
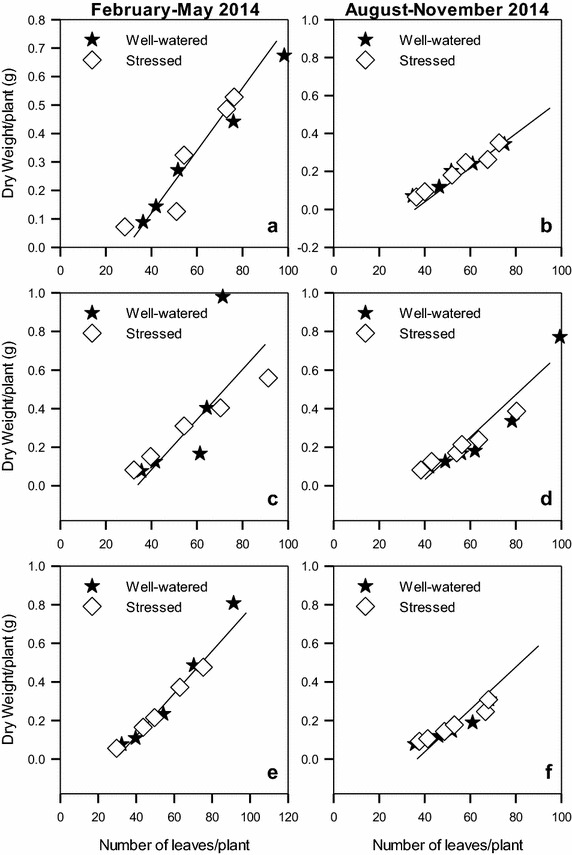
Relationship between number of leaves produced and dry weight accumulated by linseed varieties *Summit* (**a**, **b**), *S19/12* (**c**, **d**) and *Raja* (**e**, **f**) grown during the periods February–May and August–November, 2014

For the three varieties, increase in number of leaves resulted in a linear increase in dry weights (Fig. 6; Table 2). The rate of this increase was independent of the variety and was similar in both seasons. In the two seasons, a unit increase in leaf number resulted in 0.01 times increase in dry weight for all the varieties.

**Table 2:** The slope and intercepts and their standard errors for the linear functions in Fig. 6

Variety	February–May 2014					August–November 2014				
	Slope	SE	Intercept	SE	R^2^	Slope	SE	Intercept	SE	R^2^
S19/12	0.01	0.001	0.258	0.062	0.970	0.008	0.0006	0.228	0.034	0.981
Raja	0.014	0.003	0.437	0.156	0.897	0.01	0.0012	0.336	0.077	0.938
Summit	0.011	0.001	0.319	0.05	0.983	0.01	0.001	0.314	0.0079	0.927

**Fig. 7:**
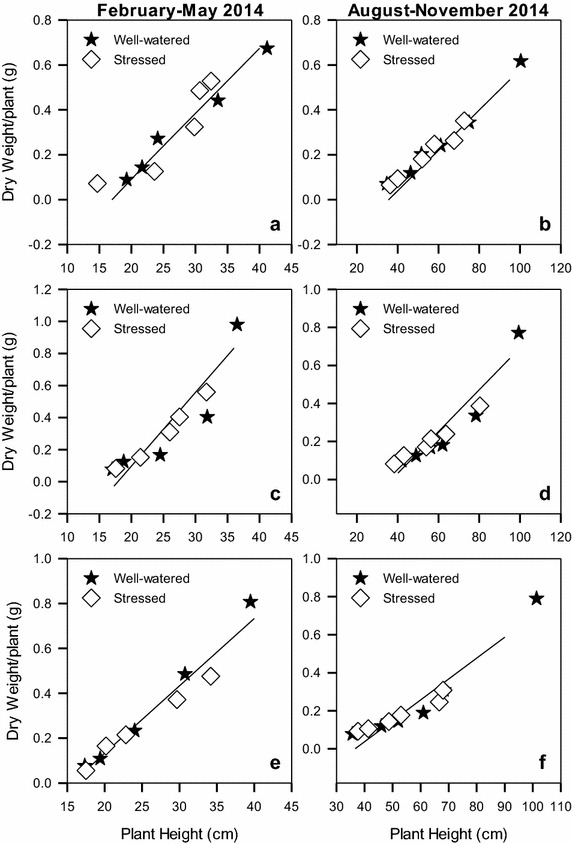
The trend of plant height versus dry weight relationships for linseed varieties *Summit* (**a**, **b**), *S19/12* (**c**, **d**) and *Raja* (**e**, **f**) grown during the periods February–May and August–November, 2014

There was a linear relationship between increase in plant height and increase in plant dry weight for all the varieties in both seasons (Fig. 7). A unit increase in plant height for S19/12 and Summit resulted in 0.028–0.030 times increase in dry weight during both seasons (Table 3). A unit increase in height for Raja produced 0.038 times increase in dry weight in both seasons. This increase was significantly higher than that for S19/12 and Summit.

**Table 3:** The slope and intercepts and their standard errors for the linear functions in Fig. 7

Variety	February–May 2014					August–November 2014				
	Slope	SE	Intercept	SE	R^2^	Slope	SE	Intercept	SE	R^2^
S19/12	0.028	0.003	0.436	0.08	0.969	0.029	0.002	0.521	0.042	0.988
Raja	0.038	0.007	0.642	0.191	0.900	0.038	0.005	0.775	0.141	0.930
Summit	0.029	0.001	0.452	0.019	0.998	0.030	0.005	0.547	0.139	0.889

**Fig. 8:**
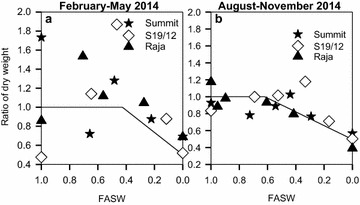
Ratio of plant dry weight of stressed plants to well watered plants in response to changing fraction of available soil water (FASW) for linseed varieties grown during the periods February–May (**a**) and August–November (**b**), 2014

Decline in the rate at which plants accumulated biomass caused by water stress during the February–May 2014 season started at 0.43 FASW (Table 1). It was however reached at 0.62 FASW during the August–November 2014 season. In both seasons, severe water stress caused plants to cease accumulating biomass. This occurred when the ratio of biomass of the stressed to well watered plants was 0.3–0.5 (Fig. 8).

**Fig. 9:**
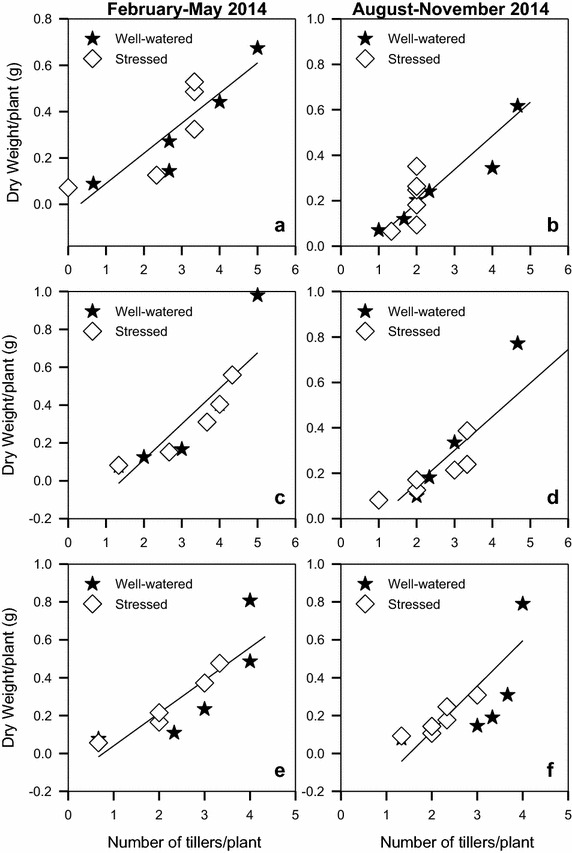
Contribution of tillering to accumulation of dry weight by linseed varieties *Summit* (**a**, **b**), *S19/12* (**c**, **d**) and *Raja* (**e**, **f**) grown during the periods February–May and August–November, 2014

There was a linear increase in plant dry weight with increase in tillers for all the varieties in both seasons (Fig. 9). A unit increase in number of tillers resulted in 0.130–0.188 times increase in dry weight; for the three varieties in both seasons (Table 4).

**Table 4:** The slope and intercepts and their standard errors for the linear functions in Fig. 9

Variety	February–May 2014					August–November 2014				
	Slope	SE	Intercept	SE	R^2^	Slope	SE	Intercept	SE	R^2^
S19/12	0.130	0.04	0.04	0.121	0.782	0.180	0.030	0.171	0.071	0.898
Raja	0.188	0.052	0.26	0.17	0.814	0.184	0.032	0.230	0.085	0.895
Summit	0.173	0.051	0.133	0.139	0.794	0.187	0.061	0.248	0.160	0.704

**Fig. 10:**
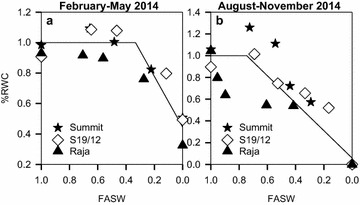
Ratio of relative water content of stressed plants to well watered plants in response to changing fraction of available soil water (FASW) for linseed varieties grown during the periods February–May (**a**) and August–November (**b**), 2014

Reduction in relative water content caused by water stress during the February–May 2014 season started at 0.33 FASW (Table 1). This decline begun at 0.75 FASW during the August–November 2014 season. As available water decreased, so did the plant’s relative water content (Fig. 10).

## Discussion

The three tested varieties did not vary in their production of leaves. Linseed has been reported to produce 60–80 leaves by Van der Voseen and Mkamilo (2007). Previous field research by Lilian et al. (2014) reported that Summit, S19/12, Raja, Jawhar and S19/12 produced 150–250 leaves with the varieties not differing significantly in the production of leaves. The current results are for a pot experiment and that is probably why the reported number of 92–100 leaves is significantly lower than the earlier number. Exposure to conditions of water stress resulted in a significant reduction in the rate of production of leaves. This could be as a result of reduced rate of leaf initiation with setting in of water stress. The decline in the rate of production of leaves was first indicated when 50 % of available water had been used up in both seasons. During both seasons, there was a relationship between production of leaves and accumulation of dry matter in the three varieties; an increase in number of leaves was accompanied by a corresponding increase in dry weight. Water stress can exert a strong influence on leaf area development by decreasing leaf appearance rate (Turay et al. 1992), duration and leaf expansion rate (Turner 1997) and increasing the rate of leaf senescence and abscission (Sinclair et al. 1984). Bazzaz and Harper (1977) found the total leaf area of linseed to be largely determined by the number of leaves and therefore concluded that a reduced leaf number due to water stress probably accounted for the low leaf area index in rain fed plants. For the three linseed varieties, production of leaves was severely affected by severe stress. A unit increase in number of leaves resulted in a linear increase in dry weight, a relationship which was similar for the three varieties. Leaves are photosynthetic sites and therefore the more the leaves the more the photosynthates are produced and accumulated in the plant. Total dry matter accumulation has been shown to be a function of assimilating organs and the photosynthetic capacity of the leaf canopy (Bisco and Gallangher 1977; Diepenbrock and Porksen 1992). In this study, water stress caused a reduction in number of leaves of 23 %.

Water stress caused as a significant decline in height of the three tested linseed varieties. Well watered plants reached heights of 39 cm. Stress produced shorter plants whose heights averaged 31 cm; this was true in both seasons. Summit and Raja varieties reached the heights of 40 cm during the February–May season. These were taller than S19/12 which grew to 36 cm high. As plant available water reduced so did the rate of plant growth in height. Hiruy and Nigussie (1988) reported that linseed growth to height of 50–80 cm. Lilian et al. (2014) reported height of 60–80 cm for five linseed varieties grown in the field. Many earlier studies on other crops such rice (Davatgar et al. 2009), maize (Muhammad et al. 2001; Porro and Cassel 1986; Hernadez 1980) and safflower (Mohammad et al. 2012) have reported reduction in plant height due to water stress which they attributed to inhibition of cell elongation or cell division. The same can be said of the three linseed varieties. In this study, a reduction of 21 % in height resulted due to water stress. There was a positive linear relationship between increase in plant height and increase in dry weight for the three linseed varieties. Similar findings have been reported by Lilian et al. (2014) for five linseed varieties planted in the field. Plant cell division causes plant elongation hence increase in plant height. As these cells mature, they have a direct contribution to increase in biomass.

 Linseed has been reported to produce 4–5 tillers (Ali et al. 2011; Mohammad et al. 2012). In the current study, water stress caused a significant reduction in production of tillers during the two seasons, of up to 25 %. Well watered plants produced 4–5 tillers which reduced to 3–4 tillers per plant for those plants where stress was imposed. There were no variations in number of tillers produced by the three varieties, though S19/12 produced a high number of 4–5 tillers in comparison to Raja which produced lowly 2–3 tillers. Earlier studies by Lilian et al. (2014) reported up to 14 tillers per plant. Studies on wheat cultivars Inqlab-91 and Uqab-2000 by Akram (2011) found significant reduction in tillers due to water stress. Imposition of water stress at the stem elongation and anthesis stage caused a reduction from 698.8 to 663.0 tillers per m^2^. A minimum reduction in tiller numbers per hill (from 19 to 18) on basmati rice varieties Basmati-Super, Shaheen-Basmati and Basmati-385 was reported by Akram et al. (2013). This was attributed to the fact that at the time of water stress, maximum tillers had been developed by the plants. In a greenhouse experiment, with two sugarcane genotypes (CP 80-1743 and CP 01-2390), Zhao et al. (2012) reported significant reduction in number of tillers due to water stress. During the 2009 experiment, the tillers ranged from 1 to 4 and 2 to 6 tillers per plant for the stressed and well watered pots respectively. The numbers rose to 5–8 and 6–10 tilers per plant for the stressed and well watered pots respectively in 2010. Gabiana (2005) reported as many as twice branches/plant in irrigated (2.5) compared to the unirrigated (1.2) linseed plants. Increase in dry weight was linearly related to increase in tillers. This is because each individual additional tiller had an accompanying biomass.

During both seasons S19/12 had higher dry weight (0.75–0.80) compared to Summit and Raja which averaged 0.60–0.66. This is perhaps from the higher number of tillers it produced during both seasons. The well-watered plants produced 0.73–0.82 g/plant of dry weights in both seasons. This was higher than that of the stressed plants, which produced 0.35–0.52 g/plant dry weights. This could have resulted from the individual direct contribution to dry weight by number of leaves and plant height, parameters which also varied significantly between the well-watered and stressed plants. As water stress affected individual parameters, the whole was translated into an effect on dry weight. The total biomass produced by a crop during its life cycle, in response to the existing environmental conditions can be defined as dry matter (Hassan and Leitch 2001). Environmental factors can indirectly influence crop dry matter production through their effect on the rate of photosynthesis and respiration (Robertson 1984). Chartzoulakis et al. (1993) reported 60–65 % reduction in dry weight in Kiwifruit cv. Hayward growing under severe water stress, in a glasshouse. Halil et al. (2001) studied the effect of water stress on eggplant (*Solanum melongena* L. cv., Teorem F1). They observed a 27–43 % reduction in dry weight under severe water stress conditions (60–40 % pot capacity) which was attributed to metabolic regulation of adaption to water stress. Total dry matter production in unirrigated plots was significantly lower than in irrigated plots throughout the life cycle of the linseed crop (Gabiana 2005). Irrigation increased dry weight by 59 % from 509 to 763 g/m^2^. In this study, as available soil water reduced, so did the of accumulation dry weight. The tested linseed varieties had only accumulated 55 % of potential dry weight by the time they completely dried.

Relative water content (RWC) reduced with decrease in available soil water for the three tested linseed varieties (Fig. 10). During the February–May season, the %RWC started to decline at 0.33 FASW while the decline begun at 0.75 FASW during the August–November season (Table 1). The RWC parameter is considered as one of the easiest agricultural parameters that can be used to screen for plants drought tolerance (Boutraa et al. 2010). Drought tolerant plant species maintained high RWC compared with drought-sensitive species in cultivars of sugarcane (Marcelo et al. 2007). Stoyanov (2005) reported that water stress causes a decrease in RWC in beans species. Tambussi et al. (2000) reported that cultivars of wheat under water stress showed a decrease in the RWC. There are many reports about the direct relationship between relative water content and drought resistance (Shimshi et al. 1982; Merah 2001; Schonfeld et al. 1988) from which deductions on ability to adjust intracellular water relations under drought stress conditions have been made. The variation in the FASWs at which  %RWC declined during the two seasons could have arisen from the differences in environmental relative humidity, which could have been higher during the August–November season than during the February–May season. This could have lowered the rates of evapotranspiration thus enabling the soil to continue holding more water for longer (data not presented). The tested linseed cultivars can be considered as drought-sensitive since, during the February–May season they maintained a very low %RWC.

## Conclusion

It was concluded that subjecting linseed varieties S19/12, Raja and Summit to permanent wilting results in reduced production of leaves, growth in height, production of tillers and dry weight by 20–40 %. Decline in all growth parameters begun when 30–80 % of available soil water had been used up. The linear relationships, which existed between the various growth parameters, were not influenced by the water status of soil. Varieties too did not influence these linear relationships. Relative water content for the three linseed varieties declined after 25–67 % of available soil water had been used up.

## Methods

This study investigated the effect of water deficit on three linseed cultivars (Summit, S19/12 and Raja) grown in the greenhouse of the department of Horticulture at the Jomo Kenyatta University of Agriculture and Technology (JKUAT), Kenya. The experiment was setup in a completely randomized design. Pots were weighed then filled with 2 kg soil obtained from JKUAT farm. Ten seeds were sowed in each pot before thinning to one after establishment. Gravimetric soil water content and field capacity for this soil were determined. Treatments were applied 4 weeks after sowing and comprised withholding water application to a half of the pots (stressed) until the plants attained permanent wilting point. The well watered control was maintained at 90 % soil field capacity (FC) throughout the experimental period by weighing the pots daily and replacing the amount of water lost. Destructive harvesting was done guided by the water levels on plants under stress; at 90, 70, 60, 50, 40 %FC and at end point. The experiment was replicated thrice and was repeated twice (February–May and August–November 2014). Data on plant height, number of leaves, number of tillers, and dry weight was recorded. Relative water content (RWC) was determined according to Turner (1986) where fresh leaves were taken from each variety at each harvest and weighed immediately to record fresh weight (FW). Then they were placed in distilled water for 4 h and weighed again to record turgid weight (TW). These were then subjected to oven drying at 70 °C for 24 h to record dry weight (DW). The RWC was calculated using the equation:

$${\text{RWC}} = \left\{ {\left( {{\text{FW}} - {\text{DW}}} \right) /\left( {{\text{TW}} - {\text{DW}}} \right)} \right\} \times 100.$$

All data were subjected to analysis of variance (ANOVA) using PROC GLM in SAS 9.1.3 portable version and means separated using LSD procedure at the 0.05 level of significance and graphs plotted using Microsoft Excel and SigmaPlot 12.0. Growth parameters were expressed as ratio of stressed to the well watered. Fraction of available soil water (FASW) was derived using a formula; FASW = 1 − {(Wc − We)/(Ws − We)} where Wc = current weight of pot, We = weight of pot at end point (permanent wilting point), Ws = weight of pot at saturation. The relationship between the ratio of a particular growth parameter and FASW was expressed as a non-linear function in SAS and then plotted in SigmaPlot 12.0. The point at which the ratio begun to decline was the fraction of available soil water critical point (FASW_c_). Linear relationships were also derived between number of leaves per plant and dry weight per plant, plant height and dry weight per plant, and, number of tillers per plant and dry weight per plant, using linear regression procedure in SAS. All these were again plotted in SigmaPlot 12.0.

## Abbreviations

ALA: alpha-linolenic acid
ANOVA: analysis of variance
CI: confidence interval
DHA: docosahexaenoic acid
DM: dry matter
DW: dry weight
EPA: eicosapentaenoic acid
FM: fresh mass
FC: field capacity
FW: fresh weight
LCPUFA: long chain polyunsaturated fatty acid
RWC: relative water content
TW: turgid weight
